# Clinic Atlanta Medical College

**Published:** 1875-10

**Authors:** A. W. Calhoun

**Affiliations:** Professor of Diseases of Eye and Ear


### Clinic Atlanta Medical College.

A. W. CALHOUN., M.D., PROFESSOR OF DISEASES OF THE EYE AND EAR.

(*Continued from page* 38 *April Journal.*)

CASE 18. *Double cataract—Extraction.*—R. D., white, æt. seventy-two years, Thomasville, Georgia. While the eyes are normal and the cataracts mature, this case is not the most favorable for an operation, inasmuch as the patient is quite old and in very feeble health. In addition to this, you perceive he has what is in common parlance known as "shaking palsy," which of itself will interfere very materially with the progress of the operation. Still, taking the condition of the patient into consideration, we must make it without an anæsthetic.

The cataract was successfully removed from the left eye by means of Graefe's linear extraction, notwithstanding the constant severe shaking of the body and the eye itself, rendered it very difficult and dangerous to bring the operation to a termination The wound healed in the ordinary time under the usual treatment after cataract operations.

Three weeks after the first operation, the extraction (Graefe's linear) was made upon the right eye. Bearing in mind the difficulty that was encountered in operating before, the patient was now etherized, and kept perfectly quiet during the entire operation, which was made without hinderance. At the end of ten days the patient was walking about the grounds, both eyes having sufficiently recovered to admit of outdoor exercise and exposure to light. Soon afterwards he was dismissed, with vision in right eye equal to 12-100 with cataract glass (double convex) No. 4, and being able to read Jæger No. 8 with double convex glass No. 2½. In left eye vision was equal to 12-50 with glass No. 4, and he could read Jæger No. 8 with glass No. 2½.

Where double cataract exists, it is, as a rule, best to wait until the one eye is out of danger from the operation before making the extraction upon the other. The object of this is simply to avoid, in the second operation, whatever circumstances may have arisen to prevent the complete success of the first, and which probably could not have been foreseen had both operations been made at once. The double operation is, however, sometimes justifiable, where it is impossible for the patient to lose the time taken up in the interval between the operations.

It is of interest to mention that in this case the complete anæsthesia not only stopped the palsy during the operation, but held it thoroughly checked for a week afterwards. The query arises, would not such treatment be beneficial in such troubles, under certain circumstances?

CASE 19. *Episcleritis*—Col'd woman, æt. thirty years, Atlanta, Georgia. We have here an extremely troublesome and stubborn though not a very dangerous affection. You recognize it by the enlargement of the sclerotic blood vessels, giving a peculiar dark purplish hue, a sort of dusty appearance, to the subconjunctival tissue. Little reddish elevations take place upon the sclerotic near the edge of the cornea. There is some redness in the conjunctiva, but you can readily see that the congested vessels are in the sclerotic, by moving the overlying conjunctiva, when the smaller blood vessels in it will be found to pass back and forth over the larger and stationary vessels of the sclerotic. There is almost always more or less photophobia, lacrimation, and some ciliary neuralgia, and an unpleasant if not painful feeling in and around the eye. It is slow to get well, and recurs again and again. In most instances it is due to a rheumatic tendency or to some syphilitic taint, and treatment directed to the cure of these troubles will have most effect in benefiting the eye. Iodide potassium with minute portions of corrosive sublimate, preparations of colchicum or guaiacum will be found beneficial, and in patients, as in the one before us, who are broken down and feeble, tonics are quite necessary. As local treatment, most good is to be derived from the use of atropine (gr. ij. to ℥) dropped into the eye several times daily, with the view of dilating the pupil and preventing any complications taking place in the iris, and of acting as a sedative to the eye. Warm applications for a short time and at intervals will give great relief in many cases. But bear in mind the stubborn character of the disease, and that it is often but little influenced by any treatment.

CASE 20. *Ectropium of both lower lids; operation by two different modes; successful.*—G. J., col'd, æt. twenty-five years, Atlanta, Georgia. The patient has been working in a rolling mill for one or two years, and has been constantly exposed to the intense heat arising from the iron furnaces. A chronic conjunctivitis was set up, and by a gradual relaxation and hypertrophy of the conjunctiva of the lower lids, and at the same time a gradual contraction of the skin upon the outer surface, the entire lids have been everted, the ciliary border of each being pulled so far down upon the cheeks that the whole of their mucous lining is exposed. You have in this a typical case of ectropium, resulting from a rather frequent cause, for it has often been observed that a continued exposure of the eyes to severe heat will not infrequently produce such a result as is here seen.

The disease being of so severe a form, it was not thought necessary to try anything but some of the operations by which it could be radically cured; and upon each lid a different operation was made, to demonstrate the good effects of the two. A long, straight incission, running parallel with and about one line from the edge of the left lower lid, was made from near the inner canthus to a point three or four lines beyond the outer. The lid was then dissected up until it could readily be replaced in its natural position. A pointed flap of skin was next taken from the left cheek, by extending the cut downwards for about one and a half inches from the outer extremity of the first incision, and at a right angle with it, and again upwards, giving the flap sufficient breadth and length and the proper shape to fit into the space left after replacing the now loosened lower lid. A broad base was left to nourish the flap. After removing the undue amount of fatty tissue and checking the bleeding and cleansing the parts, the flap was turned into its new position, the point towards the inner canthus, and a sufficient number of fine sutures applied to bring about exact apposition between the adjoining edges, and healing by first intention. The space from which the flap came was closed by bringing the edges of the wound together and uniting them by sutures. A light bandage was applied and the wounds dressed daily. At the end of the third day the sutures were removed, and the flap found firmly seated in its new position. In three weeks the patient was dismissed with the deformity completely removed, the new-formed lid being only a little thick, which should disappear after a short while, and with its motion being in perfect order, the underlying muscular fibres having been left intact.

*Operation upon right lid.*—A few weeks after the cure of the left, the following operation was made upon the right lid, for the removal of the ectropium. Two straight incisions were made through the skin, beginning at the outer and inner angles of the eye, passing down and meeting upon the front of the cheek, about an inch and a quarter below the edge of the lid, forming a triangular flap, with the base upwards and the apex downwards. Dissecting up the flap from its apex upwards, the lid could easily be replaced in its natural position; and as the lid was shoved upwards, taking the flap with it, the angle from which the apex came (the junction of the two incisions) was left uncovered. With the lid and flap held in their replaced and natural positions, the parts were stitched together, so as to insure their healing at once, and the uncovered angle below was closed by bringing the edges together also with sutures. These were removed in a few days, and after the lapse of a few weeks the cure was so perfect, together with the movement of the lid, that it was almost impossible to observe that such a disease had existed.

CASE 21.—*Pterygium—operation.*—N. P., colored; Georgia. Arlt, of Vienna, maintains that a pterygium is produced by a small abrasion or ulcer having at some time existed about the edge of the cornea, which in healing up draws a portion of conjunctiva into it, and this gradually growing and extending, produces such a trouble as you now see. The disease nearly always occurs on the nasal side of the eye, but occasionally above, below, or to the outer side. Not long since I operated upon a case in which a pterygium occupied both the inner and outer angles of the eye, the apices very nearly meeting in front of the pupil. It has a base towards the inner canthus, and the apex is fastened to the margin of the cornea, or extends over upon its surface. With a probe you can separate the new growth from the underlying conjunctival tissue, leaving only the base and apex attached.

We shall make here what is known as the needle operation, viz: passing a thread underneath the pterygium at three different points, and ligating it at its base and apex, and also the connective tissue underneath it. This strangulates the parts, which slough off after a few days, under the continued pressure of the ligatures. The conjunctiva always becomes more or less inflamed and swollen, but it passes away in a few days.

The great trouble with pterygium is that it is prone to return, notwithstanding the various operations devised to prevent it. The above mode is an old one, but I hope before long to show you a more recent plan devised and practiced by Arlt, with considerable success.

Four weeks after the above operation you see the patient free from his disease, and he is dismissed, with the request to return should the pterygium reappear.

